# Iatrogenic Obliteration of Ureter with Spontaneous Recanalization

**DOI:** 10.1155/2013/290725

**Published:** 2013-09-03

**Authors:** Darren Beiko, Ben Mussari

**Affiliations:** ^1^Department of Urology, Kingston General Hospital, Queen's University, 76 Stuart Street, Kingston, ON, Canada K7L 2V7; ^2^Department of Diagnostic Radiology, Kingston General Hospital, Queen's University, 76 Stuart Street, Kingston, ON, Canada K7L 2V7

## Abstract

We report an unusual case of spontaneous and complete healing of a severe iatrogenic midureteral injury. Following percutaneous nephrostomy and 3 months on our surgical waiting list, the injured ureter underwent complete spontaneous recanalization. The patient is clinically well with no evidence of recurrent obstruction after 2 years of followup. To our knowledge, this is the first reported case of spontaneous recanalization of an iatrogenically induced complete ureteral obliteration.

## 1. Introduction

Severe ureteral injuries involve complete ligation, transection, obliteration, or avulsion of the involved ureter [1, 2] and require open or laparoscopic surgical repair since it is usually not possible to place a ureteral stent across a completely occluded, transected, or avulsed ureter [3, 4]. We report an interesting case of spontaneous recanalization of a completely occluded midureteral injury that occurred as a result of a suture injury during a complex transabdominal urogynecologic reconstructive operation. 

## 2. Case Report

A G4P4 62-year-old female initially presented with stress urinary incontinence due to intrinsic sphincter deficiency and severe pelvic organ prolapse. Her past medical history was significant for asthma, hypertension, obesity, smoking, and chronic back pain, and her medications included salbutamol, hydrochlorothiazide, conjugated estrogens, oxycodone, meloxicam, and amitriptyline. Her past surgical history was significant for total abdominal hysterectomy and bilateral salpingo-oophorectomy (TAHBSO), Burch colposuspension, appendectomy, and cholecystectomy. Of importance, she sustained an apparent injury to the right ureter during her TAHBSO. After treatment attempts using pessaries and one failed bladder neck collagen injection, she underwent a complex transabdominal urogynecologic reconstructive operation that involved sacrocolpopexy with mesh, Halban culdoplasty, bilateral paravaginal repair, suprapubic tension-free vaginal tape (TVT) procedure, posterior repair, perineoplasty, cystoscopy, and suprapubic catheter insertion. Intraoperatively, moderate hemorrhage was noted on the right side of the pelvis, and two absorbable #1 coated Polyglactin 910 (VICRYL, Ethicon, Inc. 2007) figure-of-8 sutures were used to achieve hemostasis. There were no other complications, but the patient required transfusion of 2 units of packed red blood cells on postoperative day (POD) 1. The patient was discharged home in stable condition on POD 5.

On POD 7, she started experiencing intermittent nausea, fever, malaise, decreased appetite, and purulent drainage from her abdominal incision. Her family physician treated her wound infection with oral erythromycin and arranged daily dressing changes by the home care service. Over the next 3 weeks, she had persistent symptoms and had now lost 10 kilograms since her operation 4 weeks earlier, so she presented to the hospital for assessment. On examination, she appeared somewhat unwell, but her vital signs were stable. Abdominal examination revealed significant purulent drainage from a partly open and unhealed incision, but there were no peritoneal signs. Bloodwork was normal other than an elevated creatinine of 178 *μ*mol/L and mild hyponatremia with a sodium of 128 mmol/L. Computed tomography (CT) scan showed a draining anterior abdominal wall abscess, a 6.7 × 4.2 × 2.6 cm fluid collection underneath the abdominal wall abscess in the space of Retzius, and moderate right hydroureteronephrosis to the level of the mid ureter.

**Figure 1:**
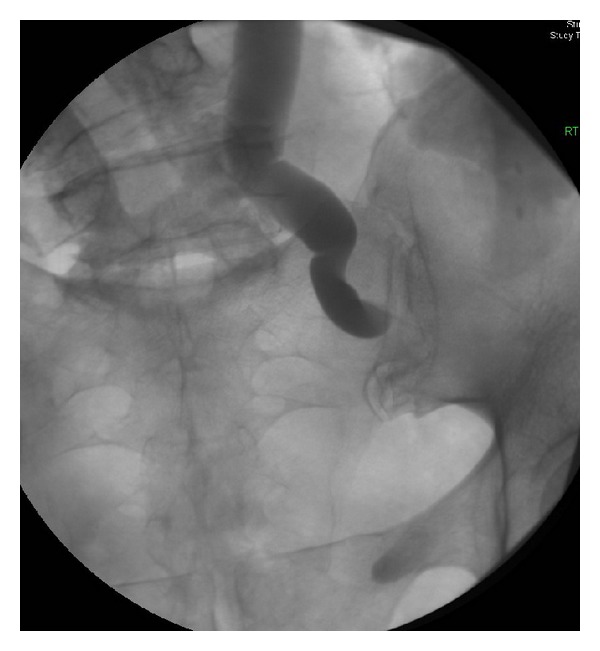
Antegrade nephrostogram showing complete obliteration of the lower end of the right mid ureter (patient in prone position).

She was admitted to hospital and was treated with a right percutaneous nephrostomy tube and intravenous antibiotics. Her wound was openly draining. Antegrade nephrostogram showed a dilated and tortuous ureter up to an abrupt and complete disruption of the ureter at the lower end of the mid ureter (Figure 1). Her clinical status improved, and her bloodwork parameters normalized. The patient was therefore discharged home 3 days later with a prescription for oral levofloxacin and metronidazole and home nursing care for her wound and nephrostomy tube.

 The patient was seen in the urology clinic 2 weeks later, now 6 weeks following her operation. Given the location and severity of the ureteral injury, all reconstructive surgical options were discussed, including ureteral reimplantation with psoas hitch and/or Boari flap and/or renal descensus, ipsilateral ureteroureterostomy, transureteroureterostomy, ileal interposition, renal autotransplantation, and nephrectomy. Informed consent was obtained, the patient was placed on the waiting list for surgery, and the nephrostomy tube remained indwelling.

**Figure 2:**
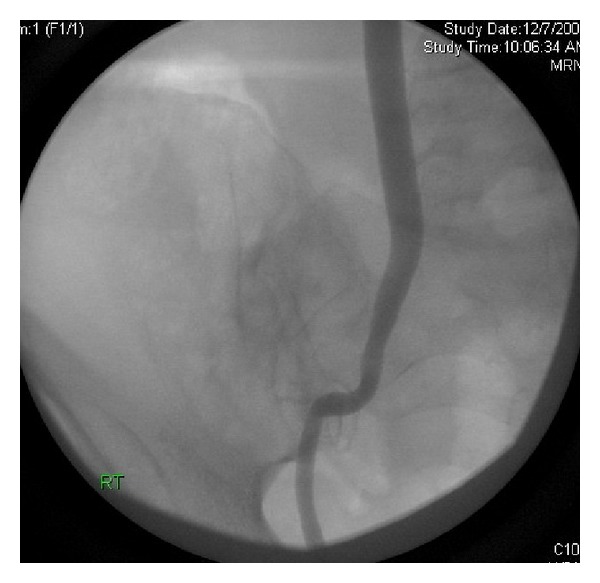
Antegrade nephrostogram showing recanalization of the ureteral lumen (patient in supine position).

Three months later, she was brought to the operating room for planned surgical repair of the injured ureter. Simultaneous antegrade nephrostogram and retrograde ureteropyelogram were performed to determine the precise length of the obliterated ureteral segment, which would in turn aid in surgical planning. Unexpectedly, the antegrade nephrostogram showed complete recanalization of the previously totally occluded midureteral segment (Figure 2). The anticipated surgical reconstructive operation was therefore cancelled, and a ureteral stent was placed in an antegrade fashion and removed a several weeks later. After 2 years of followup, the patient remains well with no urinary incontinence, lower urinary tract symptoms, prolapse, or evidence of recurrent ureteral stricture.

## 3. Discussion

The majority of ureteral injuries are iatrogenic in nature [5]. Although the reported incidence of iatrogenic ureteral injuries during gynecologic surgery varies widely from 0.05 to 30% [6], most would agree that the actual incidence is between 0.35–1% [7]. Such injuries are often known to occur most frequently during urology (42%), gynecology (34%), and general surgery (24%) cases, involving the distal ureter, mid ureter, and proximal ureter in 91%, 7%, and 2% of injuries, respectively, [8]. Risk factors for ureteral injuries during gynecologic surgery have been identified, including cancer, hemorrhage, endometriosis, adhesions, enlarged uterus, and laparoscopy [9]. Ureteral injuries may or may not be identified intraoperatively. However, intraoperative identification of a ureteral injury has been shown to have a significant effect on the ultimate treatment, the number of procedures required to treat, ultimate fate of the affected renal unit, and overall morbidity [3, 8]. Loss of renal function is very rare when such injuries are identified and treated intraoperatively [8].

 Since ureteral injuries are associated with a high morbidity, surgeons must have a high level of suspicion. Furthermore, prevention of ureteral injuries is crucial. Several prevention strategies have been reported including having a thorough knowledge of ureteral anatomy and its normal course [10], adequate surgical training and meticulous surgical techniques [11], being aware of the operation-specific regions where the ureter is most susceptible to injury [12], preoperative intravenous pyelogram or other imaging [13], and the placement of ureteral catheters or stents preoperatively [13, 14].

There are various types of ureteral injuries including ligation, crush, laceration, avulsion, stretch, and devascularization [9]. Ureteral crush or stretch injuries are best managed with an indwelling ureteral stent for 2–4 weeks [15]. When a ligation or laceration ureteral injury is identified intraoperatively, treatment depends on location of injured ureter. For injuries of the mid ureter or proximal ureter, a simple stented ureteroureterostomy is the treatment of choice as long as there is no devascularization of either end of the ureter [15]. For injuries of the distal ureter or ureterovesical junction, ureteroneocystostomy is performed [15]. 

When a ureteral injury is identified in the early postoperative period, minimally invasive approaches may be successful. For example, percutaneous antegrade balloon dilation was shown by Liatsikos et al. to result in a 60% patency rate after 1 week [16]. However, when a ureteral injury is only recognized in the late postoperative period, minimally invasive approaches often fail [17].

Complete or severe ureteral injuries are less common than minor ureteral injuries, and their management is more complex. For completely transected or obliterated ureteral injuries, it is usually not possible to place a ureteral stent across the injured segment, and therefore, conservative management often fails. Consequently, patients often require open or laparoscopic repair with appropriate urinary reconstruction involving ureteral reimplantation, psoas hitch, Boari flap, transureteroureterostomy, renal descensus, ileal interposition, renal autotransplantation, or nephrectomy [18].

There have been 2 publications reporting successful conservative management of ureteral injuries [19, 20]. In fact, in a study by Lask et al., 16 of 20 patients with ureteral injuries had complete spontaneous healing of the injured ureter after an average of 32 days (range 14–66 days). Although the authors concluded that PCN enabled spontaneous recovery of the injured ureter in the majority of patients, they acknowledged that patients with severe ureteral injuries that clearly would not resolve with percutaneous nephrostomy drainage alone were excluded [19]. 

Given the right-sided pelvic bleeding that necessitated two figure-of-8 sutures and the remoteness and lack of connection of the ureter to the fluid collection in the space of Retzius, we conclude that the sutures were the cause of the ureteral injury; the fluid collection anterior to the bladder was simply too far away to cause extrinsic compression of the ureter. Our case demonstrates the innate ability of the ureter to heal. The transformation of the completely obliterated ureter in Figure 1 to the widely patent ureter in Figure 2 over a period of 3 months illustrates this. Despite this unique case, we do not recommend that patients with iatrogenic ureteral injuries marked by a completely obliterated lumen be managed with an indwelling nephrostomy tube with follow-up antegrade nephrostogram 2 or 3 months later. However, for partial thickness ureteral injuries, indwelling percutaneous nephrostomy +/− ureteral stent may enable spontaneous ureteral recovery.

## References

[1] Best CD, Petrone P, Buscarini M (2005). Traumatic ureteral injuries: a single institution experience validating the american association for the surgery of trauma-organ injury scale grading scale. *Journal of Urology*.

[2] Akay AF, Girgin S, Akay H, Şahin H, Bircan MK (2006). Gunshot injuries of the ureter: one centre’s 15-year experience. *Acta Chirurgica Belgica*.

[3] Teber D, Egey A, Gözen AS, Rassweiler J (2005). Ureteral injuries. Diagnostic and treatment algorithm. *Der Urologe*.

[4] Trottmann M, Tritschler S, Graser A (2007). Injuries of the renal pelvis and ureter. Diagnosis and management. *Der Urologe*.

[5] Elliott SP, McAninch JW (2006). Ureteral injuries: external and iatrogenic. *Urologic Clinics of North America*.

[6] Mariotti G, Natale F, Trucchi A, Cristini C, Furbetta A (1997). Ureteral injuries during gynecologic procedures. *Minerva Urologica e Nefrologica*.

[7] Liapis A, Bakas P, Giannopoulos V, Creatsas G (2001). Ureteral injuries during gynecological surgery. *International Urogynecology Journal and Pelvic Floor Dysfunction*.

[8] Selzman AA, Spirnak JP (1996). Iatrogenic ureteral injuries: a 20-year experience in treating 165 injuries. *Journal of Urology*.

[9] Drake MJ, Noble JG (1998). Ureteric trauma in gynecologic surgery. *International Urogynecology Journal and Pelvic Floor Dysfunction*.

[10] Kim J-H, Moore C, Jones JS (2006). Management of ureteral injuries associated with vaginal surgery for pelvic organ prolapse. *International Urogynecology Journal and Pelvic Floor Dysfunction*.

[11] Rafique M, Arif MH (2002). Management of iatrogenic ureteric injuries associated with gynecological surgery. *International Urology and Nephrology*.

[12] Chan JK, Morrow J, Manetta A (2003). Prevention of ureteral injuries in gynecologic surgery. *American Journal of Obstetrics and Gynecology*.

[13] Watterson JD, Mahoney JE, Futter NG, Gaffield J (1998). Iatrogenic uketeric injuries: approaches to etiology and management. *Canadian Journal of Surgery*.

[14] Al-Awadi KA, Kehinde EO, Al-Hunayan A, Al-Khayat A (2005). Iatrogenic ureteric injuries: Incidence, aetiological factors and the effect of early management on subsequent outcome. *International Urology and Nephrology*.

[15] Fry DE, Milholen L, Harbrecht PJ (1983). Iatrogenic ureteral injury. Options in management. *Archives of Surgery*.

[16] Liatsikos EN, Karnabatidis D, Katsanos K (2006). Ureteral injuries during gynecologic surgery: treatment with a minimally invasive approach. *Journal of Endourology*.

[17] Ku JH, Kim ME, Jeon YS, Lee NK, Park YH (2003). Minimally invasive management of ureteral injuries recognized late after obstetric and gynaecologic surgery. *Injury*.

[18] Benoit L, Spie R, Favoulet P (2005). Management of ureteral injuries. *Annales de Chirurgie*.

[19] Lask D, Abarbanel J, Luttwak Z, Manes A, Mukamel E (1995). Changing trends in the management of iatrogenic ureteral injuries. *Journal of Urology*.

[20] Dowling RA, Corriere JN, Sandler CM (1986). Iatrogenic ureteral injury. *Journal of Urology*.

